# Nontyphoidal *Salmonella* Invasive Disease: Challenges and Solutions

**DOI:** 10.1093/ofid/ofad020

**Published:** 2023-06-02

**Authors:** John A Crump, Tonney S Nyirenda, Lisette Mbuyi Kalonji, Marie-France Phoba, Bieke Tack, James A Platts-Mills, Melita A Gordon, Samuel M Kariuki

**Affiliations:** Centre for International Health, University of Otago, Dunedin, New Zealand; Department of Pathology, Kamuzu University of Health Sciences, Blantyre, Malawi; Department of Medical Biology, University Hospital of Kinshasa, Kinshasa, Democratic Republic of the Congo; Department of Microbiology, Institut National de Recherche Biomédicale, Kinshasa, Democratic Republic of the Congo; Department of Medical Biology, University Hospital of Kinshasa, Kinshasa, Democratic Republic of the Congo; Department of Microbiology, Institut National de Recherche Biomédicale, Kinshasa, Democratic Republic of the Congo; Department of Clinical Science, Institute of Tropical Medicine, Antwerp, Belgium; Department of Microbiology, Immunology and Transplantation, KU Leuven, Leuven, Belgium; Division of Infectious Diseases and International Health, School of Medicine, University of Virginia, Charlottesville, Virginia, USA; Malawi Liverpool Wellcome Trust Programme, Blantyre, Malawi; Institute of Infection, Veterinary, and Ecological Sciences, University of Liverpool, Liverpool, United Kingdom; Centre for Microbiology Research, Kenya Medical Research Institute, Nairobi, Kenya

**Keywords:** antimicrobial drug resistance, bacteremia, diarrhea, epidemiology, *Salmonella*, vaccines

## Abstract

Nontyphoidal *Salmonella* are a leading cause of community-onset bacteremia and other serious infections in sub-Saharan African countries where large studies indicate that they are an uncommon cause of moderate-to-severe diarrhea. Approximately 535 000 nontyphoidal *Salmonella* invasive disease illnesses and 77 500 deaths were estimated to occur in 2017; 422 000 (78.9%) illnesses and 66 500 (85.9%) deaths in countries in sub-Saharan Africa. Lineages of *Salmonella enterica* serovar Typhimurium sequence type (ST) 313 and lineages of *Salmonella enterica* serovar Enteritidis ST11 dominate as causes of invasive disease. A major reservoir for these specific strains outside of humans has not been identified to date. Human fecal shedding of such strains is common in areas where nontyphoidal *Salmonella* invasive disease incidence is high. The case-fatality ratio of nontyphoidal *Salmonella* invasive disease is approximately 15%. Early diagnosis and treatment are needed to avert fatal outcomes. Antimicrobial resistance, including multiple drug resistance, decreased fluoroquinolone susceptibility, and resistance to third-generation cephalosporins, is increasing in prevalence and is likely to further compromise patient outcomes. Naturally acquired immunity against invasive disease develops in children aged >3 years in endemic areas, likely mediated in part by the sequential acquisition of T-cell immunity, followed by antigen-specific immunoglobulin G antibodies. Vaccines in preclinical or clinical development include live-attenuated *S. enterica* serovar Typhimurium, nontyphoidal *S. enterica* core and O-polysaccharide glycoconjugates, multiple antigen-presenting system complexes, and generalized modules for membrane antigens vaccines. The latter are in phase I trials in Europe and Africa. Both vaccine use, and other effective, evidence-based nonvaccine interventions, are needed to prevent and control nontyphoidal *Salmonella* invasive disease.

## Teaser

Nontyphoidal *Salmonella* are a leading cause of community-onset bacteremia, but not diarrhea, in sub-Saharan African countries. Research provides insights on reservoir, source and mode of transmission. Management is compromised by antimicrobial resistance. Phase I trials of vaccine candidates are underway.

Nontyphoidal *Salmonella* are a leading cause of bacteremia in sub-Saharan Africa countries [1, 2], where they also cause meningitis or focal infections, often in the absence of recent or current diarrhea [3]. Of the estimated approximately 535 000 nontyphoidal *Salmonella* invasive disease illnesses and 77 500 deaths in 2017, 422 000 (78.9%) illnesses and 66 500 (85.9%) deaths occurred in countries in sub-Saharan Africa [4]. Most disease occurs among those aged <5 years of age [4]. Immunocompromised or malnourished individuals, including those with human immunodeficiency virus (HIV) or recent malaria, are at increased risk [3, 5]. Due to its nonspecific clinical presentation, nontyphoidal *Salmonella* invasive disease is difficult to distinguish from other causes of severe febrile illness, including malaria [6], by clinical history and physical examination alone [7]. Most infections are caused by *Salmonella enterica* serovar Typhimurium or *Salmonella* Enteritidis [8]. Among these, *Salmonella* Typhimurium sequence type (ST) 313 [9] and the so-called ‘Africa-restricted clades’ of *Salmonella* Enteritidis ST11 dominate [10, 11]. Furthermore, the emergence of antimicrobial resistance to many classes of drugs [12] threatens to worsen the already very high case-fatality ratio of almost 15% associated with the disease [13].

Despite the sobering burden of disease associated with nontyphoidal *Salmonella* invasive disease in sub-Saharan Africa countries, substantial research investment to develop both vaccine and nonvaccine prevention strategies has only recently begun. With growing attention to nontyphoidal *Salmonella* invasive disease, this article summarizes themes on key challenges and solutions discussed by subject matter experts during a roundtable session at the 12th International Conference on Typhoid and Other Invasive Salmonelloses held on December 6–8, 2021 [14].

## BURDEN OF DISEASE

Although the past decade has seen the publication and improvement of burden of disease estimates for nontyphoidal *Salmonella* invasive disease [4, 15, 16], recent interest has included whether the burden of nontyphoidal *Salmonella* diarrhea in sub-Saharan African countries could strengthen the case for vaccine development and use. For example, the Institute for Health Metrics and Evaluation Global Burden of Disease 2019 (GBD 2019) study estimated approximately 50 000 annual deaths in children under 5 years of age due to nontyphoidal *Salmonella* invasive disease and 40 000 annual deaths due to nontyphoidal *Salmonella* diarrhea [17]. This would suggest a substantial combined burden that would strengthen a nontyphoidal *Salmonella* disease value of vaccine case. However, there are some reasons to question the GBD 2019 nontyphoidal *Salmonella* diarrheal disease burden estimate. The GBD estimates for etiology-specific diarrheal deaths are based on estimates of the proportion of diarrhea requiring hospitalization from multipathogen studies with molecular diagnostics that attribute deaths to specific pathogens. However, because deaths, including diarrheal disease deaths, in low-resource settings often occur without access to care [18], hospital-based studies may not be a suitable proxy to determine the causes of fatal diarrhea. To obtain their estimate, GBD used several model-based estimates to adjust studies that include any combination of enrolling children who were not hospitalized, interrogating a single pathogen, or using nonmolecular diagnostics. Because GBD 2019 estimated approximately 500 000 diarrheal deaths in children under 5 years of age, the nontyphoidal *Salmonella* diarrheal disease estimate implies that approximately 8% of hospitalized diarrhea was attributable to nontyphoidal *Salmonella*. However, primary data from rigorous diarrhea etiology research studies in children in low-resource settings suggest that the proportion of diarrhea attributable to nontyphoidal *Salmonella* might be considerably lower [19–22].

The Global Pediatric Diarrhea Surveillance (GPDS) Network began surveillance in 28 countries in 2017. From 2017 through 2018, 29 502 children under 5 years of age hospitalized with diarrhea were enrolled, with 5465 (18.5%) tested by quantitative polymerase chain reaction (PCR) for a wide range of potential causes. In GPDS, approximately 1% of diarrhea was attributable to nontyphoidal *Salmonella* using an assay that broadly detected *Salmonella enterica* [19]. Similarly, the Global Enteric Multicentre Study (GEMS), done from 2007 through 2011 in 7 countries in Africa and Asia, enrolled children with moderate-to-severe diarrhea, approximately 20% of whom were hospitalized [20]. *Salmonella enterica* was the attributable cause for slightly more than 1% of diarrhea in that study and slightly less than 2% of the subset of participants with hospitalized diarrhea [21]. Estimates of the prevalence of *Salmonella* diarrhea among diarrhea of any severity have also been low. For example, the Malnutrition and Enteric Disease (MAL-ED) study, a community-based birth cohort study in 8 diverse low-resource settings in Africa, Asia, and South America, analyzed quantitative PCR data from 6625 diarrheal stools identified by active surveillance of participants from enrollment until 2 years of age among 1715 children [23]. *Salmonella enterica* was found to be the attributable cause of less than 0.1% of diarrhea and was the 25th most common enteric pathogen identified in this study.

On balance, these data suggest that the GBD 2019 estimate of nontyphoidal *Salmonella* diarrhea deaths may be overestimated. The GBD 2020 estimates will incorporate the GPDS data and are forthcoming. Ideally, future improvements would incorporate direct estimates of causes of diarrheal deaths, irrespective of where they occur. Such estimates are not easy to obtain, but population-based child mortality surveillance studies such as the Child Health and Mortality Prevention Surveillance (CHAMPS) network may be particularly valuable [18, 24]. However, it is not immediately clear that the burden of nontyphoidal *Salmonella* diarrhea substantially strengthens the case for vaccine development and use, or whether prevention of nontyphoidal *Salmonella* invasive disease should remain the primary goal.

## EPIDEMIOLOGY

In Kenya, nontyphoidal *Salmonella* invasive disease was documented to be a major cause of severe and fatal febrile illness among hospitalized children well before the onset of the HIV pandemic [25]. Today, as in other sub-Saharan African countries, *Salmonella* Typhimurium and *Salmonella* Enteritidis account for the majority of infections. Among these, *Salmonella* Typhimurium ST313 lineages I and II and *Salmonella* Enteritidis ST11 Central and East African lineages predominate and are associated with high prevalence of antimicrobial resistance, including to third-generation cephalosporins [26, 27]. Nontyphoidal *Salmonella* invasive disease incidence in rural and urban settings in Kenya ranges from 166 to 625 per 100 000 per year [27, 28].

It has been hypothesized that humans may be an important reservoir for nontyphoidal *Salmonella* strains associated with invasive disease in Kenya and more widely in Africa [9, 11, 29]. Although researchers continue to search for and exclude nonhuman reservoirs [30], it is necessary to understand patterns of human carriage and the magnitude of human fecal shedding because this may be an important factor in transmission [31]. Recent studies in the Democratic Republic of the Congo have shown high genetic relatedness between almost 30% of nontyphoidal *Salmonella* isolates from blood with isolates from stool samples [32, 33]. In epidemiologic studies in Kenya, researchers have found a high prevalence of nontyphoidal *Salmonella* fecal shedding in populations with a high incidence of nontyphoidal *Salmonella* invasive disease. In addition, shedding continued for up to 3 months in some individuals in a crowded, informal settlement in Nairobi where water, sanitation, and hygiene challenges were common, suggesting that the human gut is likely an important reservoir for relevant strains, with human feces as a source [28]. With poor patient outcomes and growing prevalence of antimicrobial resistance, prevention of nontyphoidal *Salmonella* invasive disease by vaccine and nonvaccine interventions is an urgent priority. The latter would be informed by research to understand the role of potential sources of infection such as fecally contaminated water, food, or direct contact in transmission, and further work to identify, or to rule out, nonhuman reservoirs.

In many sites in sub-Saharan African countries, nontyphoidal *Salmonella* invasive disease is more prevalent during the rainy season [5, 34, 35]. This may be due to increased host susceptibility to nontyphoidal *Salmonella* invasive disease due to increased malaria transmission during the rainy season [36], or it could be associated with increase environmental exposure to the pathogen through fecal contamination of water and food. Research in Kisantu, Democratic Republic of the Congo, using retrospective clinical data and remote sensing climate data showed an association of nontyphoidal *Salmonella* invasive disease with rainfall that was independent of malaria [36]. This suggests a role for the environment in nontyphoidal *Salmonella* transmission and highlights the importance of environmental studies.

## TRIAGE AND EMPIRIC MANAGEMENT

Nontyphoidal *Salmonella* invasive disease typically presents as a severe febrile illness or sepsis that is difficult to distinguish from other causes of febrile illness [6]. Early identification and treatment are likely to be central to improving patient outcomes. At the first level of healthcare, healthcare workers may fail to recognize danger signs warranting prompt treatment and referral [37]. Handheld diagnostic devices, such as automated respiratory rate counters, may facilitate detection of danger signs, but they need to be adapted for use in low-resource settings [38, 39]. Because causes of severe febrile illness cannot be reliably distinguished, and coinfection is possible, World Health Organization (WHO) guidelines recommend treating all children with severe malaria with broad-spectrum antibacterials [40]. Despite the importance of early empiric antimicrobial treatment to limit the mortality of nontyphoidal *Salmonella* invasive infections, only approximately 6% of children with severe malaria have a confirmed nontyphoidal *Salmonella* bacteremia. In a number-needed-to-threat analysis, 16 patients would need to receive a broad-spectrum antimicrobial to treat 1 nontyphoidal *Salmonella* infection [41]. Research is ongoing on a clinical decision support model to better target antibacterial treatment according to the risk of nontyphoidal *Salmonella* bacteremia in children under 5 years of age admitted to Hôpital Saint Luc, Kisantu with severe febrile illness [42]. Accurate rapid diagnostic tests for nontyphoidal *Salmonella* invasive disease would greatly assist in making decisions for targeted treatment for patients.

## ANTIMICROBIAL RESISTANCE

A recent systematic review of isolates associated with nontyphoidal *Salmonella* invasive disease in sub-Saharan Africa showed that since 2001, multiple drug resistance (MDR), defined as resistance to ampicillin, trimethoprim-sulfamethoxazole, and chloramphenicol, was observed in 75% of nontyphoidal *Salmonella* isolates from all sub-Saharan African regions [12]. Third-generation cephalosporin resistance emerged in all sub-Saharan African regions and was present in 5% of isolates after 2010. Decreased fluoroquinolone susceptibility emerged in all sub-Saharan African regions but did not increase over time. Azithromycin resistance was reported in the Democratic Republic of Congo. There were no reports of carbapenem resistance [12].

Microbiologic surveillance of bloodstream infections has been in place at several sites in the Democratic Republic of the Congo since 2007, where nontyphoidal *Salmonella* has been identified as the leading cause of bacteremia with *Salmonella* serovars Typhimurium and Enteritidis dominating [35, 43–45]. Surveillance has shown that in excess of 80% of nontyphoidal *Salmonella* bloodstream isolates were MDR [12]. In addition, resistance to fluoroquinolones has emerged, and since 2011 resistance to third-generation cephalosporins has also emerged [35, 43, 45]. More recently, strains combining MDR with resistance to third-generation cephalosporins and azithromycin have been isolated. Molecular analysis found that this pattern of resistance was associated with a particular strain of *Salmonella* Typhimurium ST313 sublineage II.1 that appears to be expanding in the country [46]. Similarly, there is a long history of surveillance for bloodstream infections and antimicrobial resistance in Kenya where MDR nontyphoidal *Salmonella* has been present since at least the 1990s [10, 47]. Resistance of nontyphoidal *Salmonella* isolated from the bloodstream to third-generation cephalosporins was first reported from Kenya in 2012 [48], and decreased fluoroquinolone susceptibility has also been identified [49].

## TARGETED ANTIMICROBIAL MANAGEMENT

The antimicrobial management of nontyphoidal *Salmonella* invasive disease is challenged by widespread MDR [12]. Third-generation cephalosporins are the mainstay of empiric antibacterial management of severe febrile illness, and targeted therapy of nontyphoidal *Salmonella* bacteremia relies on fluoroquinolones and ceftriaxone to which antimicrobial resistance is becoming increasingly widespread [12].

With the increasing prevalence of MDR, deceased fluoroquinolone susceptibility, and third-generation cephalosporin resistance, azithromycin may be a candidate for oral treatment of uncomplicated disease or for oral switch based on efficacy for management of uncomplicated typhoid fever [50]. Research has determined that minimum inhibitory concentration and zone diameter epidemiologic cutoffs established for *Salmonella* Typhi are also valid for nontyphoidal *Salmonella* invasive disease [51, 52]. However, pharmacokinetic and pharmacodynamic studies, and the collection of clinical outcome data, are needed to establish clinical breakpoints. Clinical data are crucial because, compared with typhoid fever, nontyphoidal *Salmonella* bacteremia presents more frequently with sepsis, and higher extracellular antimicrobial concentrations may be needed for nontyphoidal *Salmonella* than for *Salmonella* Typhi bacteremia [53, 54]. Ongoing research in Hôpital Saint Luc, Kisantu, Democratic Republic of the Congo is collecting clinical and microbiologic outcome data in children under 5 years of age admitted with nontyphoidal *Salmonella* invasive disease [55]. Results to date identified problems with accurate dose calculation and administration due to limited availability of good quality and age-appropriate formulations in this low-resource setting.

## IMMUNITY

In sub-Saharan African countries, infants, young children aged <2 years, and, to a lesser extent, neonates are particularly susceptible to nontyphoidal *Salmonella* invasive disease [56]. The peak of disease occurrence is observed from 12 through 18 months of age, and robust protective naturally acquired immunity against invasive disease has developed in children aged above 3 years in endemic areas. This is likely mediated in part by the sequential acquisition of T-cell immunity, followed by antigen-specific IgG antibodies [57]. Antigen-specific IgG antibodies targeting lipopolysaccharide inversely correlate with age-stratified disease incidence and also mediate functional serum bactericidal immunity against nontyphoidal *Salmonella* [56]. Children who lack serum bactericidal immunity are susceptible to nontyphoidal *Salmonella* invasive disease, and this supports vaccine interventions to confer protection among young, susceptible children [57].

Malaria is associated with increased risk for nontyphoidal *Salmonella* invasive disease in malaria-endemic settings [58]. Malaria causes global impairment of immune components including phagocytes, serum immunity, and T-cell immunity [59, 60]. In malaria-endemic settings, malaria induces immune defects, particularly in serum bactericidal immunity, thereby compromising the performance of antibody vaccines against nontyphoidal *Salmonella* [59]. Malaria disease has been implicated in depleting complement proteins that are essential for serum bactericidal activity, and this occurs regardless of nontyphoidal *Salmonella*-specific IgG antibody titers [59]. Ongoing studies in Malawi will further investigate these observations at scale. To optimize future vaccines and antibodies, these studies seek to further characterize immunogenic antigens and immunity in healthy children and children with comorbidities including malaria, anemia, and malnutrition.

## VACCINES

Vaccines in preclinical or clinical development include live-attenuated *S. enterica* serovar Typhimurium, nontyphoidal *S. enterica* core and O-polysaccharide glycoconjugates, multiple antigen-presenting system complexes, and generalized modules for membrane antigens (GMMA) vaccines, derived by membrane ‘blebbing’ from *S. enterica* serovars Enteritidis and Typhimurium. GMMA vaccines have the potential advantage that multiple polysaccharide and membrane protein antigens are expressed in their natural configuration on outer membrane vesicles. Phase 1 studies of GMMA vaccines are underway in Europe and Africa, and others will soon follow [61–64]. An optimal vaccine to prevent nontyphoidal *Salmonella* invasive disease should, at minimum, protect against *Salmonella* serovars Typhimurium and Enteritidis that are responsible for the majority of human infections in African countries [13]. Vaccines will also need to protect individuals from early in life [8] and those with comorbidities such as HIV, malaria, and malnutrition, making it particularly important to understand the correlates of susceptibility and protection in these key groups. The WHO has held recent expert landscape and stakeholder consultations on *Salmonella* vaccines [65]. These included consideration of the possible benefits of developing multivalent *Salmonella* vaccines that might protect against a wider range of both typhoidal and nontyphoidal *Salmonella* serovars, to improve programmatic efficiency and effectiveness [61]. The emergence of somatic antigen O:5-negative *Salmonella* Typhimurium in the Democratic Republic of the Congo is a possible cause for concern for O-antigen-based vaccines in development, and it underlines the importance of continuous surveillance for the emergence of strains that might escape or evade vaccine protection [43].

## CONCLUSIONS

Nontyphoidal *Salmonella* are a major cause of community-onset bacteremia and other serious infections in many countries in sub-Saharan Africa. Disease is common early in life and among those with HIV, recent malaria, and malnutrition. On the other hand, nontyphoidal *Salmonella* do not appear to be a major cause of severe child diarrhea in the same settings. *Salmonella* Typhimurium ST313 and *Salmonella* Enteritidis ST11 account for most infections. Humans may be an important reservoir for relevant strains. Prolonged fecal shedding is common both after infection and disease in affected communities. Studies in nonhuman animals have been limited. However, to date, they have failed to identify a major nonhuman reservoir. Sources and predominant modes of transmission have not been studied in detail, but fecally contaminated water and food are likely to play a role. Understanding the chain of transmission comprehensively will be central to nonvaccine prevention.

The case-fatality ratio of nontyphoidal *Salmonella* disease is estimated to approach 15%. Patient outcomes are likely to be further compromised by lack of early recognition and effective treatment. Antimicrobial resistance is a major concern in nontyphoidal *Salmonella* strains causing invasive disease, with MDR, decreased fluoroquinolone susceptibility, and resistance to third-generation cephalosporins widespread in some area.

Although protective immunity against nontyphoidal *Salmonella* invasive disease is seen in children aged above 3 years mediated in part by antigen-specific IgG antibodies and T cells, comorbidities such as malaria, anemia, and malnutrition pose a challenge to protecting children in endemic areas. Phase 1 studies of GMMA vaccines are underway and other candidates are in development. To optimize future vaccines and antibodies, ongoing studies seek to further characterize immunogenic antigens and immunity in healthy children and children with comorbidities. In addition, attention should be paid to serovar variation and the emergence of O-negative variants. Both vaccine use and other effective, evidence-based, nonvaccine interventions are needed to prevent and control nontyphoidal *Salmonella* invasive disease.
